# A Case of Chronic Granulomatous Disease Masquerading As Tubercular Lymphadenitis in an Infant

**DOI:** 10.7759/cureus.64069

**Published:** 2024-07-08

**Authors:** Vesta Snigdha Hasa, Sanjay Kumar Sahu, Chinmay Kumar Behera, Pratap K Jena, Sarbeswar Pradhan

**Affiliations:** 1 Pediatrics, Kalinga Institute of Medical Sciences, Bhubaneswar, IND; 2 Paediatrics, Kalinga Institute of Medical Sciences, Bhubaneswar, IND; 3 Pediatrics, Kalinga Institute of Medical Sciences, Cuttack, IND; 4 Health Care Management, Swiss School of Business and Management Geneva, Geneva, CHE; 5 School of Public Health, Kalinga Institute of Industrial Technology Deemed to be University, Bhubaneswar, IND

**Keywords:** nadph oxidase, hematopoietic stem cell transplant, dihydrorhodamine test, chronic granulomatous disease, primary immunodeficiency disease

## Abstract

Chronic granulomatous disease (CGD) is a rare inborn error of immunity characterized by recurrent fungal and bacterial infections due to defective nicotinamide adenine dinucleotide phosphate (NADPH) oxidase activity. This case report describes an 11-month-old female who was initially diagnosed with tubercular lymphadenitis and presented with fever and bilateral neck swelling. Despite receiving anti-tubercular treatment (ATT) and intravenous antibiotics, the patient experienced recurrent infections and abscesses, prompting further investigation. Laboratory tests revealed normal immunoglobulin levels but abnormal nitroblue tetrazolium (NBT) and dihydrorhodamine (DHR) tests, indicating CGD. Genetic analysis (clinical exome by next-generation sequencing) confirmed a novel *NCF2* gene mutation associated with autosomal recessive CGD. This patient was treated with prophylactic antibiotics and antifungals and subsequently underwent successful hematopoietic stem cell transplantation (HSCT). This highlights the diagnostic challenges associated with CGD, particularly in tuberculosis-endemic regions such as India, emphasizing the importance of considering primary immunodeficiency disorders in patients with recurrent infections. Early diagnosis and appropriate treatment, including HSCT, can significantly improve patient outcomes. The patient remained infection-free on prophylactic antimicrobials for 1.5 years post-discharge, demonstrating the potential for a favorable prognosis with timely intervention and comprehensive management.

## Introduction

Inborn errors of immunity (IEIs) refer to a heterogeneous group of disorders characterized by poor function of one or more components of the immune system [1]. With the exception of immunoglobulin A (IgA) deficiency, other IEIs are less prevalent in regions where consanguineous marriage is not common. The clinical presentation of IEIs is highly variable, ranging from recurrent childhood infection to multiple autoimmune and inflammatory diseases, and hence can remain undiagnosed in the early stages. Chronic granulomatous disease (CGD) is a congenital IEI that presents with recurrent fungal and bacterial infection characterized by the formation of multiple systemic granulomas, which are common features of the disease. It is caused by defects in one of the subunits of the nicotinamide adenine dinucleotide phosphate (NADPH) oxidase system, resulting in the failure of the oxidative killing of catalase-positive organisms despite normal phagocytosis [2]. It is an X-linked or autosomal recessive (AR) disorder that is usually diagnosed by an abnormal dihydrorhodamine (DHR) test and is confirmed by genetic studies [3]. We encountered a very interesting patient in our hospital who was initially diagnosed with tubercular cervical lymphadenitis and subsequently diagnosed with CGD.

## Case presentation

**Figure 1:**
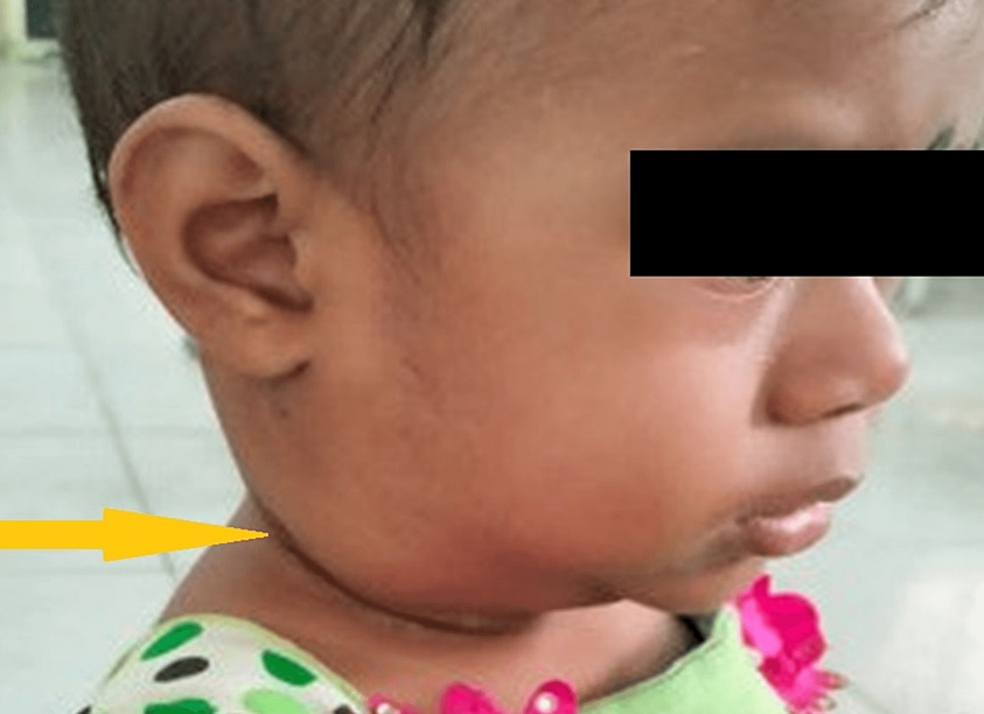
Right-sided neck swelling at the time of first hospital admission, non-erythematous with smooth surface.

An 11-month-old female infant was brought to the hospital by her mother with chief complaints of fever for 15 days and bilateral swelling of her neck for three days. She was apparently normal two weeks prior, after which she developed a fever of high grade (104°F) associated with chills and rigors. The mother noticed bilateral neck swelling for the past three days, which gradually increased in size. There was no complaint of vomiting, loose stools, skin rash, jaundice, cough or cold, or poor appetite. She was previously hospitalized twice for sepsis requiring intravenous (IV) ceftriaxone and amikacin for treatment. She was a term baby born by normal vaginal delivery out of a nonconsanguineous marriage, with a birth weight of 3.3 kg; she cried immediately after birth and had no history of (H/O) neonatal illness. Her elder sister died at 1 year of age due to liver disease, without any detailed evaluation. The mother had a history of asthma. On physical examination, the child had bilateral cervical lymphadenopathy (right side: 3×3 cm, left side: 2×3 cm), which was firm in consistency with no local rise of temperature or tenderness (Figure 1).

She had pallor, without any icterus, cyanosis, clubbing, edema, and joint swelling. Systemic examination revealed splenomegaly of 3 cm in length below the left costal margin that was firm in consistency and had rounded margins without any hepatomegaly. She was provisionally diagnosed with acute cervical lymphadenitis and was investigated. A complete blood count revealed a hemoglobin (Hb) level of 10 g/dL, a total leucocyte count (TLC) of 16,200 per cubic mm (neutrophil: 31%, lymphocyte: 61%, monocyte: 5%), a total platelet count of 246,000 per cubic mm, and normal liver and kidney function tests, with positive sepsis markers (C-reactive protein, CRP: 151 mg/L and serum procalcitonin: 2.87 ng/mL) as given in Table 1.

**Table 1:** Laboratory investigations of patients during first and second admission at our hospital

Test	Patient Value (1^st^ Admission)	Patient Value (2^nd^ Admission)	Reference Range	Unit
Hemoglobin (Hb)	10	9.1	11.1-14.1	g/dL
Total leucocyte count (TLC)	16,200	33,700	6-11 × 10^3^	/cubic mm
Neutrophil (N)	31	61	15-35	%
Lymphocyte (L)	61	32	45-75	%
Monocyte (M)	5	6	3-6	%
Eosinophil (E)	3	1	0-3	%
Total platelet count (TPC)	2,46,000	3,46,000	150-400 × 10^3^	/cubic mm
C-reactive protein (CRP)	151	136.5	0-5	mg/L
Procalcitonin	2.87	1.2	0-0.5	ng/mL
Blood urea	22	19.9	12-42	mg/dL
Serum creatinine	0.31	0.23	0.1-0.4	mg/dL
Serum sodium	137	132	136-146	mmol/L
Serum potassium	4.9	4.57	3.5-5.1	mmol/L
Serum albumin	4.6	3.4	3.5-5.5	g/dL
Serum glutamic oxaloacetic transaminase (SGOT)	45	46.8	0-40	IU/L
Serum glutamate pyruvate transaminase (SGPT)	23	39	5-40	IU/L
Serum complement (C_3_)	-	110	90-180	mg/dL
Serum complement (C_4_)	-	21	10-40	mg/dL
Total IgG	-	774.3	650-1600	mg/dL
Total IgM	-	230.99	50-300	mg/dL
Total IgA	-	69.19	17-400	mg/dL
Total IgE	-	46.76	0-46 ( for 0-3 years)	IU/mL

**Figure 2:**
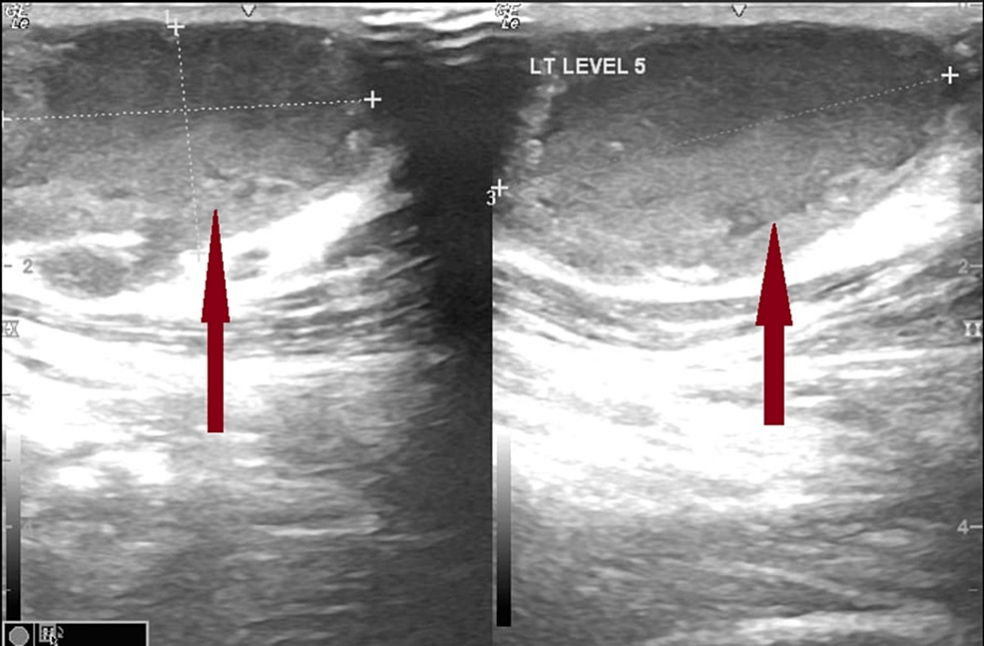
Ultrasound of neck showing bilateral conglomerated necrotic lymph nodes.

Chest X-ray was normal in appearance. The Mantoux test was non-reactive. Inj. piperacillin-tazobactum and Inj. vancomycin were started along with syrup paracetamol, as the patient had received five days of Inj. ceftriaxone outside and the treatment was continued for seven days. Her neck ultrasound (USG) showed bilateral multiple discrete and conglomerated lymph nodes with loss of fatty hilum, and necrosis without differentiation, suggestive of tubercular lymphadenitis as shown in Figure 2.

Blood culture yielded no growth. Fine needle aspiration showed few epithelioid granulomas along with sheets of neutrophils and degenerated cells, histiocytes, and lymphocytes in a dirty background. Even after seven days of IV antibiotics, she was still febrile. In view of granulomatous changes in lymph nodes characterized by epithelioid cell granuloma with central caseous necrosis, lymphocytes without any plasma cells, the various causes like lymphoma, sarcoidosis, toxoplasmosis, brucellosis, and fungal infection were excluded clinically and with appropriate investigations. Pulmonary consultation was obtained for suspected tubercular lymphadenitis, and anti-tubercular treatment (ATT) was recommended. After seven days of ATT, our patient improved clinically and became afebrile. She was discharged in a hemodynamically stable state and had normal blood parameters with ATT and proper dietary advice.

**Figure 3:**
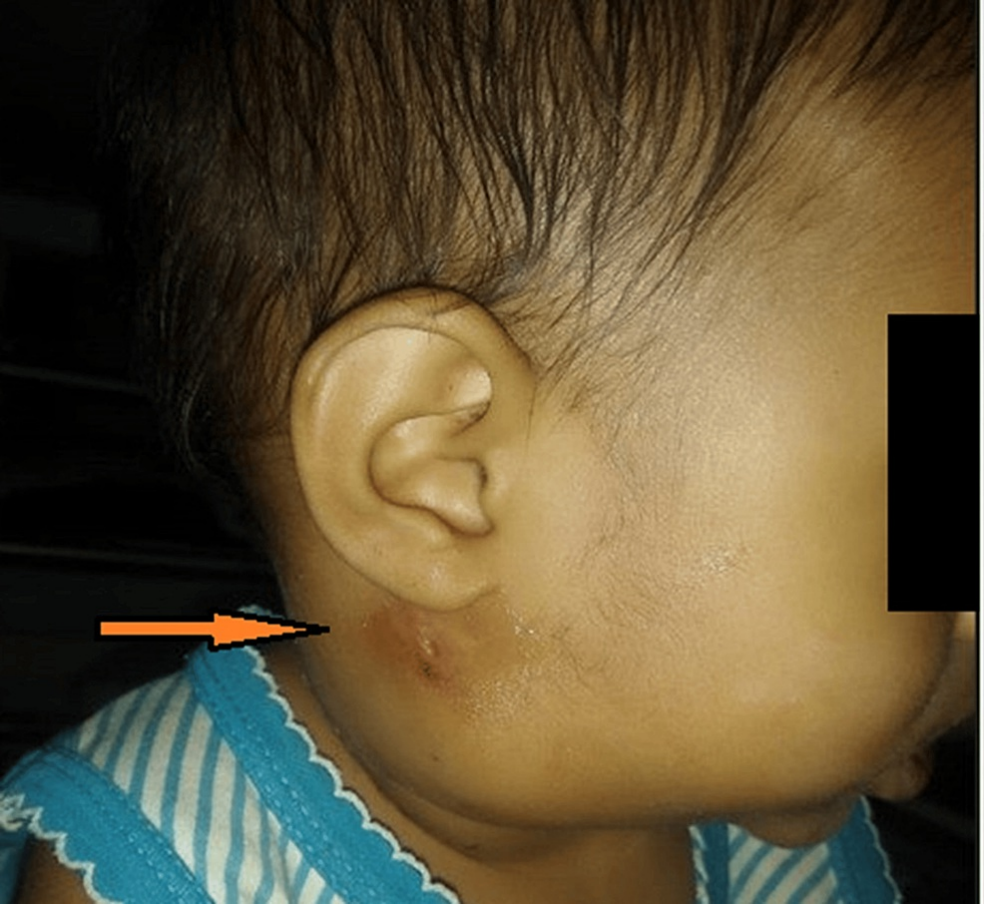
Swelling at the time of the second admission: erythematous, tender, and overlying skin shiny.

Three weeks later, she was again brought to the hospital with complaints of fever for 10 days and right-sided neck swelling for eight days. However, this fever had an insidious onset, was high grade in intensity and intermittent in nature, and responded to medication. Swelling of the right side of the neck progressively increased in size and was associated with pain. On examination, the swelling was tender and erythematous, and the skin over the swollen area was shiny and warm to the touch (Figure 3).

Relevant investigations for lymphadenitis were repeated. The patient was positive for sepsis markers (TLC: 33,7000 per cubic mm with N: 61%, L: 32%, M: 6%; TPC: 346,000 per cubic mm; CRP: 136.5 mg/L, PCT: 1.2 ng/ml), had a low Hb concentration (9.1 g/dL), and had normal serum biochemical and urinalysis results. Two sets of peripheral blood samples were collected and sent for culture. She was given Inj. amoxicillin-clavulanate, which was later upgraded to Inj. linezolid as methicillin-resistant *Staphylococcus aureus* (MRSA) was isolated from blood culture due to the persistence of fever. Despite six days of antibiotics, fever spikes persisted with a gradual increase in neck swelling, severe tenderness, warmth feeling, and redness, suggestive of abscess. A pediatric surgery consultation was obtained, and the abscess was drained along with a biopsy of the neck lymph node. Biopsy suggested reactive hyperplasia of the lymph node. Pus culture revealed *Burkholderia cepacia*, for which oral levofloxacin and doxycycline were added on the eighth day of hospitalization. The child developed vaginal candidiasis on the 12th day after admission. clotrimazole cream was applied locally.

As the child had recurrent infections caused by catalase-positive organisms (*S. aureus* in the family of Staphylococcaceae, and *B. cepacia* in the Burkholderiaceae-rRNA group II of *B. cepacia* complex) requiring multiple courses of IV antibiotics, Candida infection, evidence of deep-seated abscess during the past 1 year, the possibility of IEI (Jeffery Modell Foundation criteria: (1) requiring IV antibiotics to clear the infection, (2) two deep-seated infections - subcutaneous abscess and septicemia, and (3) vaginal candidiasis beyond 1 year of age) was considered. Specifically, CGD was suspected, for which appropriate investigations were ordered. Her human immunodeficiency virus (HIV) serology was negative. Immunoglobulin profiling, flow cytometry, nitroblue tetrazolium test (NBT), and DHR test were performed. Immunoglobulin (IgG: 774.3 mg/dL, IgM: 230.99 mg/dL, and IgA: 69.19 mg/dL, IgE: 46.76 IU/ml) and complement levels (C3: 110 mg/dL, C4: 21 mg/dL) were normal. Peripheral smears showed normal morphology of all cells. Flow cytometry revealed that all subsets of lymphocytes and NK cells were normally distributed. The NBT test of the patient did not show any change in color, and the DHR test was suggestive of the absence of neutrophilic respiratory bursts from the patient’s serum, consistent with CGD. This finding was confirmed by genetic analysis, which revealed a novel mutation (c.373T>G(p.Tyr125Asp)) of the *NCF2 *gene was found to be a variant of interest that was associated with CGD in an AR pattern (Table 2).

**Table 2:** Clinical exome analysis of the child NCF2: neutrophil cytosolic factor 2; OMIM: Online Mendelian Inheritance in Man

Gene (Transcript)	Location	Variant	Zygosity	Disease (OMIM)	Inheritance	Classification
NCF2(-) (ENST00000367535.8)	Exon 4	c.373T>G (p.Tyr125Asp)	Homozygous	Chronic granulomatous disease - 2 (OMIM#233710)	Autosomal recessive	Uncertain significance

Syrup cotrimoxazole (trimethoprim-sulfamethoxazole) (trimethoprim 5 mg/kg/day in two divided dosages) and itraconazole (5 mg/kg/day single dose) were added as per the protocol along with the continuation of ATT. She became afebrile after five days on the 25th day after admission. Proper counseling was provided to parents regarding the risk of multiple infections in the future, and the need for bone marrow transplantation (BMT) or stem cell transplantation as definitive treatment. The child was regularly followed up and remained well on antibiotic and antifungal prophylaxis for 1.5 years after discharge. Our patient had undergone allogenic (11/12 matched family donors) hematopoietic stem cell transplantation (HSCT) at the age of 2.5 years, with a myeloablative conditioning regimen. The patient had an episode of gastro-intestinal graft-versus-host disease following HSCT and was treated appropriately. The patient is now doing well at the last follow-up without any lymphadenopathy.

## Discussion

CGD was initially called ‘fatal granulomatous disease of childhood’, when it was first described in an infant in early 1950 but was not well characterized as a separate clinical entity until 1959 [4]. The incidence varies from 1 to 3 per 200,000 live births depending upon the ethnicity [5]. Initial reports of CGD from India date back to late 1990, using the NBT test [6]. The majority of CGD cases are diagnosed in early infancy, particularly below 5 years, due to recurrent bacterial and fungal infections, as in this case. The X-linked CGD, accounting for 70% of cases, is diagnosed at an earlier age than the AR-type, which constitutes the remaining 30% of cases, although a large study in India reported that AR-CGD was more common than the X-linked type in their study [7]. Compared with AR-type, X-linked diseases have earlier presentations with greater severity but with a similar mortality pattern.

A diagnosis of IEI (earlier known as PID - primary immunodeficiency) should be suspected in a patient, with recurrent pneumonia and/or ear, sinus, and cutaneous infection, as laid by The Jeffrey Modell Foundations 10 warning signs of primary immune deficiency [8]. Other important signs of IEI are excessive inflammatory responses, allergies, eczema, and autoimmunity in children. Our index patient was a female, although males are affected twice as often as females are due to the predominance of X-linked inheritance globally [9]. The X-linked recessive form of the disease caused by mutations in the *CYBB* gene accounts for 60% of patients with CGD, whereas NCF2 mutations are detected in 15% of patients from a study in India, as found in this child [7]. Defects in the NADPH oxidase complex lead to poor production of reactive oxygen species (ROS) and superoxide anions (O2-), which results in impaired killing of intracellular microorganisms, especially those that are catalase-positive. This predisposes the patient to recurrent bacterial and fungal infections. Patients with CGD usually present with pneumonia followed by abscess (superficial and deep-seated), lymphadenitis, septicemia, osteomyelitis, and meningoencephalitis [7].

Our patient had caseating granulomatous lymphadenitis, staphylococcal sepsis (MRSA), and *B. cepacia* infection isolated from a subcutaneous abscess and vaginal candidiasis. Mycobacterial disease has been reported in CGD patients, due to reduced clearance of mycobacteria (catalase-positive) and uncontrolled inflammation caused by impaired oxidative burst in macrophages [10]. In India, which is an endemic country for tuberculosis, it is not uncommon to receive empirical ATT before diagnosis, as occurred in a study by Rawat et al., where 54% of their children with persistent pneumonia had received ATT [7], and in another case report, the author described the receipt of ATT in a toddler for right submandibular lymphadenitis [2]. Hence, pediatricians in developing countries need to keep in mind that persistent pneumonia and lymphadenitis may be the initial presentations of CGD. There are reports of Bacille Calmette-Guérin (BCG)-itis infection in regions of the world where the BCG vaccine is routinely given at birth [11]. S. aureus was the most common bacteria isolated from blood, followed by Burkholderia and other gram-negative bacteria in a large study from India [7], which was also observed in this patient. Invasive fungal infection is the most common cause of death in CGD caused by Aspergillus, Mucor, and Candida species [12]. Initially, the NBT test was used as an in vitro qualitative assay to diagnose CGD [2]. However, currently, the test of choice is the flow cytometry-based DHR test, which is more sensitive than NBT and helps in differentiating among X-linked defects, autosomal defects, and CGD carriers [3]. Abnormal DHR results should always be confirmed by genetic analysis, as in our case. Long-term prophylactic antibiotics (trimethoprim-sulfamethoxazole: 5 mg/kg/day) and antifungals (itraconazole: 5 mg/kg/day) are the cornerstones of the management of CGD patients once the diagnosis is confirmed, as it reduces mortality by preventing various infections and complications. She was discharged on prophylaxis and was doing well without any breakthrough infection. The role of interferon-gamma (IFN-γ) in the current scenario is still controversial, and it is not available in all places across the world [13,14]. However, steroids, along with antimicrobial agents, play a definite role in select patients with severe inflammation [13]. Allogenic HSCT is the only curative treatment for CGD (>90%) with a high success rate if performed early, which helps to prevent infection and inflammation and reduces the use of prophylactic medication, resulting in a survival rate of up to 90% at 10 years [13]. Our patient received HSCT from a matched donor and is currently doing well after 6 months without any prophylactic medications. Gene therapy is an attractive alternative for patients without an HLA-matched donor [14,15].

## Conclusions

This case highlights the diagnostic challenges associated with CGD, a rare IEI. CGD should be considered as a differential diagnosis in children with recurrent infections, especially in tuberculosis-endemic regions. The role of genetic testing and advanced immunological assessments in achieving accurate diagnoses and effective management of CGD is of paramount importance. The patient’s favorable outcome post stem cell transplantation demonstrates the potential for a good prognosis with timely intervention. This case is a reminder that simple cervical lymphadenitis can present as CGD, mimicking tuberculosis.
